# COVID-19 Pandemic and Student Reading Achievement: Findings From a School Panel Study

**DOI:** 10.3389/fpsyg.2022.876485

**Published:** 2022-05-18

**Authors:** Ulrich Ludewig, Ruben Kleinkorres, Rahim Schaufelberger, Theresa Schlitter, Ramona Lorenz, Christoph König, Andreas Frey, Nele McElvany

**Affiliations:** ^1^Center for Research on Education and School Development (IFS), TU Dortmund University, Dortmund, Germany; ^2^Department of Educational Psychology, Goethe University Frankfurt, Frankfurt, Germany

**Keywords:** reading comprehension, reading achievement, COVID-19, elementary school, achievement gaps, large-scale assessment

## Abstract

Since 2020, the COVID-19 pandemic had an impact on education worldwide. There is increased discussion of possible negative effects on students’ learning outcomes and the need for targeted support. We examined fourth graders’ reading achievement based on a school panel study, representative on the student level, with *N* = 111 elementary schools in Germany (total: *N* = 4,290 students, age: 9–10 years). The students were tested with the *Progress in International Reading Literacy Study* instruments in 2016 and 2021. The analysis focused on (1) total average differences in reading achievement between 2016 and 2021, (2) average differences controlling for student composition, and (3) changes in achievement gaps between student subgroups (i.e., immigration background, socio-cultural capital, and gender). The methodological approach met international standards for the analysis of large-scale assessments (i.e., multiple multi-level imputation, plausible values, and clustered mixed-effect regression). The results showed a substantial decline in mean reading achievement. The decline corresponds to one-third of a year of learning, even after controlling for changes in student composition. We found no statistically significant changes of achievement gaps between student subgroups, despite numerical tendencies toward a widening of achievement gaps between students with and without immigration background. It is likely that this sharp achievement decline was related to the COVID-19 pandemic. The findings are discussed in terms of further research needs, practical implications for educating current student cohorts, and educational policy decisions regarding actions in crises such as the COVID-19 pandemic.

## Introduction

Since the beginning of 2020, the COVID-19 pandemic has led to a substantially new situation for education systems. To contain the spread of the virus that causes COVID-19, schools in many countries around the world have partially or completely closed, learning groups have been rearranged, and students or teachers had to be absent from school for various amounts of time (cf., Woessmann et al., 2020; Meinck et al., 2022). Teachers had to carry out learning activities without the usual face-to-face lessons, learners had to self-regulate at home, and parents had to support their children’s learning more than before. How these learning conditions affected students’ achievement is of considerable interest for educational policy, administration, and practice. This is especially true for reading literacy, a key competence that influences students’ achievement in other subjects and enables them to participate in society throughout their entire life course. Additionally, there is reason to assume that the COVID-19 pandemic had a differential effect on students. Even within a given education system, certain groups of students might have been affected more severely than others.

In Germany, the sudden shift from face-to-face instruction to more technologically mediated interaction and emergency remote education (ERE) was especially hard. ERE required German schools and teachers to catch up in terms of the digitalization process in education, which had been shown to lag behind other countries in the years prior to the pandemic (cf., Voogt and Roblin, 2012; Eickelmann et al., 2019; Lorenz et al., 2021). Studies have repeatedly shown that teachers lacked pedagogical skills related to technology and that students had problems accessing and using technological devices during the COVID-19 pandemic (e.g., Huber and Helm, 2020; Reimers and Schleicher, 2020; Rožman et al., 2022). Therefore, Germany might have had particular problems in adapting to the pandemic schooling situation.

A variety of recent publications have shown that schools, instruction, and stakeholders—school administrators, teachers, students, and parents—were only partially prepared for a crisis with substantial restrictions on school life such as the COVID-19 pandemic (e.g., Huber et al., 2020). Accordingly, teachers as well as parents subjectively perceived a decline in student learning (Dong et al., 2020a; Rožman et al., 2022). In contrast, some studies based on student reports found (tendentially) positive learning experiences compared to usual instruction, but students pointed out that they felt more uncertain about estimating their learning status (e.g., Huber and Helm, 2020; Rožman et al., 2022). However, there is a lack of country-specific results related to effects of the COVID-19 pandemic on key achievement measures *via* standardized tests. Highly aggregated results show that school closures due to COVID-19 had an effect of about *d* = −0.08 (Hammerstein et al., 2021) and *d* = −0.17 (König and Frey, 2022) on average student achievement across subject areas, grades, and countries. Data for Germany regarding achievement in one domain that is generalizable to a well-defined student population is missing so far.

Elementary school, and fourth grade in particular, is a pivotal moment in students’ educational biographies. At this point, reading literacy should be developed to the point where students can acquire further knowledge through reading in all subjects and continue their educational biography through independent learning. Additionally, in most federal states in Germany, after 4 years of compulsory elementary education (Grades 1–4 in age-homogenous classes of 21 students on average; Destatis, 2018), typically starting at age 6, students finish elementary school and go on to secondary schools of different tracks (Lohmar and Eckhardt, 2015). At the end of elementary school, studies before the COVID-19 pandemic repeatedly indicated that disadvantaged student groups exhibit lower reading literacy (e.g., Mullis et al., 2017). The COVID-19 pandemic might pose further risks for successful education, especially for disadvantaged student subgroups.

Taken together, students’ achievement level in important areas (e.g., reading) is of special interest after a long period of restrictions related to the COVID-19 pandemic. Additionally, whether achievement differences between student subgroups are currently greater than before is an important research question. To provide reliable comparative information on key competences before and during the COVID-19 pandemic, the present study examined reading achievement among fourth graders in German elementary schools. In this study, samples representative for the student population of all fourth graders in Germany were examined in the same 111 elementary schools in 2016 and 2021. Both samples were tested with the reading achievement tests from the international school achievement comparison study *Progress in International Reading Literacy Study* (PIRLS). We accounted for changes in student composition and investigated achievement means and how achievement gaps have evolved.

## Reading Achievement

The acquisition of reading literacy is key for further learning in other school subjects and students’ subsequent educational and life paths (Savolainen et al., 2008). Reading achievement is a core component of reading literacy, along reading motivation and behavior. In international achievement studies such as PIRLS, reading achievement represents students’ ability to extract relevant information from narrative and informational texts and to understand, use, and reflect on written texts in areas of life that are relevant to the individual and required by society (Mullis et al., 2015). Reading achievement involves multiple levels of text comprehension: surface structure, text base, situation model, rhetorical structure, and pragmatic communication (Kintsch, 1988; Graesser and McNamara, 2011). Mastering text comprehension requires sufficient word recognition (e.g., decoding skills; Wang et al., 2019), language comprehension (e.g., verbal reasoning), and bridging processes (e.g., vocabulary knowledge; see Kim, 2020), as well as active self-regulation, motivation, and engagement (Duke and Cartwright, 2021).

In the first years of schooling, students learn to read at the letter, word, and sentence level in the sense of automating reading and propositional comprehension processes. By the end of fourth grade, which is the end of elementary school in most German federal states, students are expected to comprehend increasingly longer and more complex texts (e.g., Fitzgerald et al., 2015) and to build situation models for age-appropriate texts.

There are important differences concerning comprehension of narrative and informational texts when it comes to different subprocesses (e.g., Ozuru et al., 2009). However, for pragmatic reasons, many comparative studies report on global reading achievement (e.g., Mo, 2019) that reflects comprehension of narrative and informational texts as well as other genres.

## Reading and the Impact of the COVID-19 Pandemic

Various factors must be considered in ascertaining whether and to what extent reading achievement has been affected by the restrictions related to the pandemic. Students learn to read *via* formal school-based instruction, including homework, and in their leisure time through informal reading activities. The transition from face-to-face instruction in school to ERE because of the COVID-19 restrictions led to less time for formal school-based instruction (Reimers and Schleicher, 2020). In addition, there was less instructional time available in ERE, so that overall students spent less time on learning than they would have in school (Woessmann et al., 2020). In Germany, compared to before the time spent on learning activities dropped by 62% and 42% during the first and second lockdown phases (spring 2020 and autumn/winter 2020/2021), respectively (Woessmann et al., 2020; Werner and Woessmann, 2021). At the same time, students’ leisure time behavior partly changed during ERE (Grewenig et al., 2020; Woessmann et al., 2020): the time spent on reading activities, creative work, and exercise stayed on a comparable level during the school closures in Germany (spring 2020: +11%; autumn/winter 2020/2021: −14%). But the time spent on screen-based activities such as watching TV, gaming, social media, and online media increased by a notable 21% (spring 2020) to 34% (autumn/winter 2020/2021). Children from non-college-educated households spent 1 h more on such screen-based activities than children from college-educated households (Woessmann et al., 2020). The reduction in total time spent on formal and informal reading activities and the shift toward more screen-based activities may have affected students’ achievement in reading.

Besides these substantial reductions in learning time, reading development could be negatively affected by the reduced effectiveness of instruction during the pandemic. Reading instruction could have been hampered by limited experience with technical equipment necessary for digital instruction and learning during ERE (e.g., Reimers and Schleicher, 2020; Rožman et al., 2022). This problem had been recognized in Germany even before the COVID-19 pandemic (e.g., Lorenz et al., 2021). Compared to other subjects, there are less rigorous curricular frameworks and less readily available exercises, instruction, and materials for reading teachers when reading is done (in part) at a distance (Maldonado and De Witte, 2020). Additionally, fourth graders are confronted with informational texts that involve new challenges, for instance, an increasing amount of instructional pictures (e.g., graphs, maps, and diagrams). This new challenge of cognitively demanding integrated text-picture comprehension might be difficult for teachers to support in distance learning situations (McElvany et al., 2012; Hochpöchler et al., 2013).

Currently, there is no differentiated picture of student achievement, and particularly of elementary school children’s reading achievement, during or after the restrictions related to the COVID-19 pandemic compared to before the pandemic. Several publications have already dealt with the effects of the COVID-19 pandemic on students in terms of wellbeing, school achievement, and their interactions (e.g., Hammerstein et al., 2021; Rose et al., 2021; Sánchez Amate et al., 2021). Different approaches were pursued, including a focus on theoretical considerations (e.g., Schneider et al., 2021), teacher surveys (e.g., Reimers and Schleicher, 2020; for Germany: McElvany et al., 2021), and parent surveys (e.g., Reimers and Schleicher, 2020; Steinmayr et al., 2021).

In a first systematic review on student achievement across multiple countries and grades, Hammerstein et al. (2021) focused on the effects of school closures related to COVID-19 on the subjects of math and reading. They reported heterogeneous effect sizes (*d* = −0.37 to *d* = 0.25) across studies, with a small negative effect (median *d* = −0.08) on average. These results for the first lockdown phase were corroborated by two meta-analyses. König and Frey (2022) reported an average impact of *d* = −0.12 of later school closures (after summer 2021) on average student achievement. Storey and Zhang (2021) found an effect of *d* = −0.15 across domains. Furthermore, Zierer (2021) found an average effect of *d* = −0.17 for elementary school students. Among studies examining reading achievement in elementary school children, two studies (Depping et al., 2021; Gore et al., 2021) reported very small positive effect sizes (*d* = 0.00 to *d* = 0.04). In contrast, the four studies finding negative effects on reading achievement reported larger but still small effect sizes (Engzell et al., 2021: *d* = −0.09; Maldonado and De Witte, 2020: *d* = −0.29; Schult et al., 2021: *d* = −0.07; Tomasik et al., 2020: *d* = −0.37). However, it is not yet known how the situation during the COVID-19 pandemic affected reading achievement in elementary school in Germany as a whole.

## Reading Achievement Gaps

International large-scale assessments of student achievement have repeatedly shown that Germany has some of the most pronounced social disparities (Hußmann et al., 2017; Reiss et al., 2019). There are several theories offering explanations for gaps in achievement related to family background and student variables such as gender (e.g., primary and secondary effects: Boudon, 1974; Grätz and Wiborg, 2020; expectancy-value approaches: Wigfield and Eccles, 2000; Guo et al., 2015; cultural theory: Bourdieu, 1983; and motivation as mediator: Wang and Finch, 2018; Steinmayr et al., 2021). When examining the relationship between family background and reading achievement, studies often refer to socio-cultural capital and the immigration background. Additionally, reading achievement and reading motivation are known to be systematically related to gender (Wigfield et al., 2016).

### Family Background

Children with different family backgrounds experience different levels of support from home and their reading socialization varies accordingly. Following the home literacy model (Sénéchal and LeFevre, 2002), such support may involve different literacy experiences, for instance shared reading between parents and children, teaching the alphabet, or reading words. These literacy experiences explain children’s growth in reading and vocabulary knowledge (e.g., Becker et al., 2010). Among other factors, these home literacy experiences could explain that the reading achievement of children and adolescents in Germany and many other countries is systematically associated with family background characteristics, such as socio-cultural capital or immigration background (Mullis et al., 2017; for Germany: Wendt and Schwippert, 2017).

#### Socio-Cultural Capital of the Family

Socio-cultural capital describes the social assets of a person (e.g., intellect and education). More highly educated parents are often able to support their children better and promote their children’s reading socialization more comprehensively, due to their own educational experiences and by being educational role models (Dong et al., 2020b). Therefore, higher socio-cultural capital is positively associated to reading achievement.

The number of books at home has become a frequently used indicator to approximate socio-cultural capital in large-scale assessments (e.g., Schwippert, 2019). There are large differences in reading achievement between children from families with different amounts of books at home in many countries (international: Mullis et al., 2017). In Germany, children from families with more than 100 books at home have substantially higher reading achievement on average, than children from families with a maximum of 100 books at home (Hußmann et al., 2017). There are different mechanisms that could explain these differences. (1) More books at home represent an opportunity for children to engage in reading. (2) Parents with more books are more likely to read by themselves, making them positive role models. (3) Furthermore, they are probably able to support their children to a higher degree. (4) The presence of books indicates parents’ appreciation for reading and intellectual stimulating activities and (5) is associated with a relatively stable, wealthy and spacious living situation. In sum, the amount of books at home represents a broad indicator for a family background with favorable conditions for becoming a good reader.

#### Immigration Background

On the one hand, families from immigrant backgrounds often place high value on and strongly promote their children’s education, as suggested by the immigration optimism hypothesis (Kao and Tienda, 1995). On the other hand, an immigrant background can also represent a challenge, as it is often confounded with a lower socioeconomic status, a lack of experience with the education system in the host country, and a different family language than the language of instruction, which is associated with children’s lower language skills on average (Kristen and Dollmann, 2012; Mullis et al., 2017). Immigrant parents often do not speak the language of instruction as well as native speakers, so their children may not learn the language implicitly to the same extent as their classmates, which could also affect their reading skills. This is supported by the results of PIRLS 2016, where children who always or almost always spoke German at home scored substantially higher on average than children who never or almost never spoke German at home (Wendt and Schwippert, 2017; for an in-depth longitudinal analysis, see Kigel et al., 2015).

Prior to 2021, Germany underwent a number of societal developments that have affected education. One such development is an increase in the number of immigrants coming to Germany. In 2020, about 24 percent of people living in Germany had an immigrant background. Among 5–10 year-old, 38.8 percent of children have a primary or secondary immigration background. This proportion increased by 2.7 percentage points compared to 2019 (Destatis, 2021).

### Gender

Several theoretical approaches have attempted to explain gender differences in reading achievement (for an overview of gender differences in reading and language, see Eagly and Wood, 1999; Hyde, 2014). For example, socio-cultural theory explains differences based on societal stereotypes regarding reading and learning activities (Schunk and Zimmerman, 2006). According to social-cognitive learning theory, the gender gap in reading can be explained by girls’ better self-regulatory abilities and their higher self-efficacy (cf., Hyde, 2014; McElvany et al., 2017). Additionally, reading achievement is substantially related to reading motivation (Toste et al., 2020). On average, girls have higher reading motivation and read more often in their leisure time (Ainley et al., 2002; Wigfield et al., 2016; Lepper et al., 2021), which promotes their reading achievement. Thus, a wealth of studies indicate that girls have a higher level of reading achievement than boys on average (Logan and Johnston, 2010; Mullis et al., 2017). The PIRLS 2016 results for Germany showed that fourth grade girls scored systematically higher than boys; the achievement gap favoring girls in Germany was about the same as the average achievement gap in the EU and OECD countries overall (McElvany et al., 2017).

## Reading Achievement Gaps and the Impact of the COVID-19 Pandemic

To date, there is no clear evidence on how the restrictions related to COVID-19 influenced reading achievement gaps among elementary school students. It is possible that the COVID-19-related restrictions had differential effects for different subgroups of students and therefore exacerbated educational inequality. Generally, the aforementioned achievement differences related to students’ socio-cultural capital, immigrant backgrounds, and gender can be expected to hold for the COVID-19 pandemic period as well. In fact, they may be even more pronounced because school-based support was difficult during full or partial school closures and children’s learning was left in the hands of families to a greater extent than before the pandemic (e.g., Huber and Helm, 2020). For students with lower socio-cultural capital and/or from immigrant backgrounds, the need for greater parental involvement in the learning process might have led to widening achievement gaps. As described above, parents with more socio-cultural capital are more engaged and provide more support for their children’s learning (Dong et al., 2020b). Therefore, it seems plausible that children from these families might benefit from spending more time learning with their parents. With respect to immigrant families, if learners speak a language other than the language of instruction at home, they may receive inadequate support in the language of instruction, which is particularly important for reading achievement and might have therefore affected educational outcomes in this domain during or after the COVID-19 pandemic (see Maldonado and De Witte, 2020). ERE was associated with additional costs if families had to purchase technological devices for their children to participate in the digital lessons. This may have further disadvantaged students from low-income families (Eickelmann et al., 2019; Wrase, 2020). Regarding gender, a widening achievement gap might be expected, as female students tend to have higher reading motivation and more frequently read for pleasure than male students (e.g., McElvany et al., 2017; Mullis et al., 2017). A decline in extrinsic school-based reading motivation during the COVID-19 pandemic may have led to these gender differences playing a greater role in reading improvement, which could exacerbate gender achievement gaps in the current cohort of students. Empirical evidence has shown that students’ leisure time behavior changed during the COVID-19 pandemic (e.g., Woessmann et al., 2020; Werner and Woessmann, 2021), which could affect the trends in achievement gaps. Students with more highly educated parents spent less time on leisure activities detrimental to learning than their peers and more time on conducive activities (Grewenig et al., 2020; Woessmann et al., 2020). First evidence by Engzell et al. (2021) shows a 40% larger learning loss among students from poorly educated families compared to children from highly educated families in the Netherlands.

## Current Study and Research Aim

The COVID-19 pandemic affected many areas of education, resulting in a need for empirical research how students’ learning was affected during this time. First studies indicate negative effects on students’ learning outcomes and learning behavior due to the COVID-19 restrictions. More differentiated results on reading achievement among German elementary school students are lacking so far.

The aim of this study is to provide more differentiated results on trends in elementary school students’ reading achievement by applying rigorous methodological standards and using data from a school panel study. Differences in reading achievement across different cross-sectional cohorts may be explained by changes in student composition, even when the same schools participate. Thus, the present study also controlled for changes in the student composition within each school. Furthermore, the development of reading achievement gaps during the pandemic was investigated. The students examined in this study are representative for fourth graders in Germany. We used the reading achievement tests from PIRLS 2016.

The research questions and hypotheses investigated are as follows:

1. How does the average reading achievement of fourth grade elementary school students in Germany differ in 2021 compared to before the COVID-19 pandemic in 2016?
  *H1*: Due to theoretical considerations on the impact of COVID-19-related restrictions on schooling, we expect a decline in average reading achievement from 2016 to 2021.
2. How does the average reading achievement of fourth grade elementary school students in Germany differ in 2021 compared to before the COVID-19 pandemic in 2016 after controlling for student composition?
  *H2*: We expect a decline in average reading achievement from 2016 to 2021 even when adjusting for student composition.
3. Considering achievement gaps between subgroups of students, (3a) to what extent do differences in reading achievement exist across student subgroups (socio-cultural capital, immigration background, and gender) in 2021 and (3b) how do these gaps differ in 2021 compared to 2016?
  - There is a gap in average reading achievement to the disadvantage of students with lower socio-cultural capital (H3.1.1) and this gap is larger in 2021 than in 2016 (H3.1.2).
  - There is a gap in average reading achievement to the disadvantage of students from immigrant backgrounds (H3.2.1) and this gap is larger in 2021 than in 2016 (H3.2.2).
  - There is a gap in average reading achievement to the disadvantage of boys (H3.3.1) and this gap is larger in 2021 than in 2016 (H3.3.2).

## Materials and Methods

### Participants

The target population for the school panel analyses was the cohort of fourth graders attending a general education German elementary school (i.e., one that does not cater exclusively to special education students) that existed in both 2016 and 2021 (i.e., excluding closed and newly founded schools). The analysis was based on the responses of *N* = 2,208 fourth grade students in 2016 and *N* = 2,082 fourth grade students in 2021 from a panel of *N* = 111 general education schools (with one class per school participating). All schools participated in PIRLS 2016 and were examined again 5 years later for the school panel study. Participation in the reading achievement test was mandatory in both years. Students required parental consent to fill out the student background questionnaire. Students with intellectual or physical disabilities (e.g., blindness or deafness) and recently immigrated children with less than 1 year of German instruction were free to participate but were excluded from the data set.

Data collection in 2021 was slightly affected by the COVID-19 pandemic and took place four to 6 weeks later in the school year than in 2016 (May 2 to June 3, 2016, vs. June to July 3, 2021). The absence rate on the test day was slightly higher in 2021 compared to 2016 (6.03% in 2016 vs. 9.01% in 2021). In 2021, at the time of the study, students were required to stay home at the first sign of illness. We will discuss possible consequences for the interpretation of the results later.

#### Sampling Procedure

PIRLS 2016 followed a two-stage (i.e., sampling first schools and then classes within schools) stratified cluster design (Martin et al., 2017). In 2016, a total of 208 schools were randomly sampled from a complete list of elementary schools in Germany, considering strata regarding school type (e.g., general education vs. special education schools) and the proportion of students from immigrant backgrounds as well as the additional condition that at least one school from each German Federal State had to participate. In 2021, 116 schools were sampled for the panel study as a random sample of the original *N* = 208 schools in PIRLS 2016, considering the strata school type and proportion of children from immigrant backgrounds. For the analysis, we excluded special education schools (*n* = 5) because they are structurally very different from general education schools (i.e., much smaller classes, less bound to state-mandated curricula, and students do not transition to secondary schools after fourth grade). This resulted in a sample of *N* = 111 general education elementary schools.

#### Weights

The overall weights were calculated to adjust for clustered sampling (i.e., at the school level), the combination of school, class and student weights, as well as non-response adjustment at each level (Martin et al., 2017). On average, each student in our sample from 2016 represented 294 students in the target population for 2016, and each student in our sample from 2021 represented 325 students in the target population for 2021. The 2016 sample represented 648,297 and the 2021 sample 677,762 students.

### Instruments

#### Reading Achievement Test

The reading achievement test used in PIRLS consisted of six narratives and six informational texts and different comprehension tasks developed for them (Mullis et al., 2015; Martin et al., 2017). In 2016, 181 items were administered across 15 different test versions, with each student answering items about two texts. The reading achievement test in 2021 was a subset of 120 items of the test in 2016, spread over eight different booklets. Each student answered 28.31 items on average (*SD* = 4.70) in 2016 and 27.24 items on average (*SD* = 4.50) in 2021. The items were a mixture of multiple-choice (MC) and constructed response (CR) items. The MC items were scored as either correct or incorrect. CR items were rated by trained personnel from the study administration based on scoring rubrics, as either incorrect, partially correct, or completely correct. Omitted items were scored as if they were incorrect responses and not reached items were treated as if they were not administered. The overall scoring procedure was the same in 2016 and 2021. More details on test construction can be found in Martin et al. (2017).

#### Student Composition Variables

All of the following variables are based on questions asked in both cycles (i.e., 2016 and 2021) with the same phrasing, at a similar location in the questionnaire, to the same group of respondents (i.e., students, teachers, parents, and school administrators). For binary variables, we chose a coding that sets the majority group (>50%) to 0 and the minority group (<50%) to 1, unless indicated otherwise.

##### Gender

The gender variable was based on administrative data indicating students’ gender as reported in official documents. We used contrast coding for gender, because there is no majority group (1 = Male; −1 = Female). A third category (i.e., “Other”) was only collected in 2021 and not in 2016 and could therefore not be considered in the analysis.

##### Age, Enrolment, and Grade Retention

We aimed at comparing same-aged students in 2016 and 2021. Generally, students’ age within and across cohorts of fourth graders in Germany is biased by school enrolment deadlines in Germany’s federal school system (i.e., the deadlines by which students have to turn 6 years old in order to enroll in first grade in a given year vary from August 5 to September 30 across different federal states). Additionally, the average age of participating students is higher in 2021 due to the fact that the survey period shifted slightly toward later in the school year. Furthermore, individual students’ age in fourth grade depends on whether they enrolled in school late or early relative to their birth date, and whether they were held back a grade during elementary school. Generally, being older relative to the rest of a cohort could be a developmental advantage, whereas late enrolment and grade retention are negatively associated with achievement (e.g., Bell et al., 2009). Based on these considerations, we used three variables to control for age-related aspects:

1. Relative cohort age: Students’ age within a cohort in a federal state, excluding individual deviations from regular enrolment (i.e., enrolment at age 6) and excluding grade retention. This variable represents a child’s age if all federal states had the same enrolment deadline and excludes age shifts of entire years caused by irregular enrolment and grade retention. This age variabe had a range of 1 year.
2. Enrolment: Individual deviations from regular school enrolment in years (regular enrolment is at age 6; deviations would include enrolment at age 5 or 7).
3. Grade retention: Individual deviations in the number of years of schooling in years (regular is four).

##### Immigration Background

We chose to define immigration background in three different ways based on the students’ responses.

1. The student was not born in Germany (=1) vs. the student was born in Germany (=0).
2. One or both of the students’ parents were not born in Germany (three-level factor with both parents born in Germany as the reference group: both parents born in Germany, one parent not born in Germany, and both parents not born in Germany). Place of birth for both the mother and father had to have been filled in; otherwise, the variable was set to missing.
3. The student’s family almost never or never speaks German at home (=1) vs. the family almost always or always speaks German at home (=0).

##### Socio-Cultural Capital

We used students’ responses regarding the number of books at home to approximate their cultural capital. The first group included students who reported that their families owned 100 books or less (=1) vs. students who reported that their families possessed more than 100 books (=0).

##### Special Educational Needs

In Germany, students with special educational needs have been diagnosed by an official institution as having a disability that necessitates special learning support. Specific disorders regarding scholastic skills such as dyslexia do not qualify a student for special educational support. We distinguish students with no special educational needs (=0) from students with diagnosed special educational needs (=1).

### Procedure

PIRLS 2016 and the 2021 panel study were administered by the International Association for the Evaluation of Educational Achievement (IEA) in Hamburg. Both studies were conducted entirely on paper and took place during the first half of the school day. The study was administered by trained test administrators in each class, assisted by a teacher known to the class. The test administrators were university students from related disciplines (teacher training, educational science, and psychology) who attended a mandatory workshop on international testing guidelines and the standardized testing manuals.

The testing procedure was structured the same in both cycles. First, students worked on the PIRLS achievement test in two 40-min blocks with a 10-min break in between. During these blocks, students were allowed to ask questions to clarify the instructions but not regarding how to solve the tasks. Second, after another break, students completed several further standardized tests (for cognitive ability, decoding, vocabulary, and sentence comprehension). The cognitive ability test was administered with different variations in the two cycles (e.g., different time constraints), and different instruments were used to assess the reading subprocesses, so we did not use them for the analyses presented here. Lastly, to obtain background information, students completed a questionnaire that took 45 min for PIRLS 2016 and 60 min for the panel study 2021. However, the fact that the questionnaire was longer in 2021 was not relevant to our analysis because all the questions we were interested in (immigration background and socio-cultural capital) were at the beginning of the questionnaire. In total, the study took 138 min in 2016 and 160 min in 2021, mainly because of the longer questionnaire at the end of the study.

### Data Analysis

Data preparation and analyses were performed using *R* Studio Version 4.0.3 (R Core Team, 2020). First, we used multi-level imputation to treat missing data in the background variables. Second, we scaled the test data using a multi-group IRT model. Third, plausible values were drawn based on the imputed background variables for conditioning. Fourth, we used linear mixed-effects models to examine our research questions.

#### Missing Values and Multiple Imputation

We used multiple imputation to address missing values occurring in our data. All student composition variables are based on either administrative data (e.g., age and gender) or students’ responses (e.g., books at home and immigration background). For administrative variables, the missing rate was very low, <1%. In 2016, about 10% and in 2021, about 12% of student responses on the background questionnaire were completely missing (i.e., mostly due to missing parental consent). Missing student responses were not systematically clustered within classes.

The multiple imputation was carried out separately for 2016 and 2021 with the same variables and specifications. In addition to student composition, we included parents’ reported number of books at home from the parent questionnaire and city size as auxiliary variables. For the imputation, we used a two-level imputation with predictive mean matching at level one for continuous variables (e.g., age). Furthermore, we used predictive mean matching for level two variables (i.e., city size) and logistic regression for binary variables (i.e., immigration background) within the *R* packages *miceadds* (Robitzsch et al., 2017) with 20 iterations and 10 imputed datasets.

#### Scaling and Plausible Values

Scaling for the reading achievement test was performed using a multi-group generalized partial credit model (Van der Linden, 2016). The model was estimated using the marginal maximum likelihood method (MML) with the *R* package *TAM* (Robitzsch et al., 2019). The model estimates a difficulty and a discrimination parameter for each item or response category. Prior to model estimation, we excluded two items because fewer than 5% or more than 95% of responses were correct (i.e., leaving 179 items for 2016 and 118 for 2021). The slopes within each CR item with multiple response categories were set to be equal to each other. We used a multi-group approach instead of separate scaling with linking because the achievement tests and test procedures in 2021 and 2016 were very similar. All items had a root mean squared deviation (RMSD) <0.08, so that none of the items indicated large misfit (Köhler et al., 2020). Because the item fit was acceptable for all included items, we considered the multi-group approach to be appropriate. The EAP reliability was good at *REL_EAP_* = 0.87. For all analyses, we used 10 plausible values to provide a measurement error-adjusted and unbiased estimation of effects. Plausible values were drawn using item parameters anchored at their estimated values from the calibration and random draws from the marginal posterior of the latent distribution for each student (Monseur and Adams, 2009). We used all student composition and auxiliary variables as well as their interaction with the cycle (2016 vs. 2021) for conditioning. We performed five draws with each of the 10 sets of imputed conditioning variables, resulting in 50 data sets. Finally, we used a scale that sets the mean and SD in 2016 to 1,000 and 100, respectively, to make the results of the reading achievement test easier to interpret.

#### Analysis

Proportions, means, and SDs were calculated with multiple imputed variables, overall student weighting and school clustering using the *R* package *survey* (Lumley, 2020).

Students’ reading achievement was statistically modeled using a linear mixed-effects model framework in the *R* package *lme4* (Bates et al., 2014) with the weights for 2016 and 2021. We estimated three models: (1) a gross differences model (i.e., without student composition) to compare the overall difference between the study cycles (2016 vs. 2021) and a (2) net differences model that considered changes in student composition. Additionally, we estimated (3) an achievement gap model that considers possible changes in the achievement gaps.

#### Models

First, we modeled the reading achievement (θ*_pc_*) of a student *p* = 1, …, *N* in school *c* = 1, …, *C* using a linear mixed-effect model (Bates et al., 2014). In the gross model (GM), reading achievement was modeled as a function of an intercept β_0_ (i.e., the average reading achievement in 2016), the fixed effect of the year β*_cycle_* (0 = 2016, 1 = 2021), and the random intercept of the school ζ*_c_* [the variance of ζ*_c_* was normally distributed with ζ*_c_* ~ *N* (0, σ^2^ζ)]. Thus, in our GM, β_0_ represented the average reading achievement in 2016 and β*_cycle_* the difference between 2021 and 2016.


$$GM:\theta_{pc}=\beta_{0}+\beta_{cycle}+\zeta_{c}$$

Second, the net model (NM) included all student composition variables (**X***_pk_*), *k* = 1, …, *K* as fixed effects β*_k_*. In the NM, β_0_ represented the expected average reading achievement of the reference group across cycles. The reference group represented the majority groups (born in Germany, both parents born in Germany, speaking German at home, more than 100 books at home, and no special educational needs) with average age and regular enrolment and without grade retention. The regression coefficient β_cycle_ represented the reading achievement difference between the cycles if the students’ composition and the fixed effect β*_k_* of the student composition variables were the same in both cycles.


$$\mathrm{NM}:\theta_{pc}=\beta_{0}+\beta_{cycle}+\sum_{k=1}^{K}\beta_{k}X_{pk}+\zeta_{c}$$

Third, the achievement gap model (AM) included an additional interaction between student composition and cycle. As in the other models, in the AM, β_0_ represented the reading achievement of the reference group in 2016. β*_cycle_* represents the difference between the reference group in 2016 and 2021. The interaction effect represents the difference in the deviation between the reference group and the student subgroup in 2016 vs. 2021.


$$\mathrm{AM}:\theta_{pc}=\beta_{0}+\beta_{cycle}+\overset{K}{\underset{k=1}{\sum }}\beta_{k}X_{pk}+\overset{K}{\underset{k=1}{\sum }}\theta_{k}X_{pk}*\mathrm{cycle}+\zeta_{c}$$

## Results

### Descriptive Statistics

Descriptive statistics for reading achievement are reported in Implications: Research, Support, Educational Policy, Appendix A. The student composition changed statistically significantly between 2016 and 2021, with (a) a slightly higher relative cohort age in 2021 due to later test administration dates in 2021 (*t* = 14.13, *p* < 0.001), (b) a higher percentage of children enrolled in school after turning age 6 (*t* = 2.59, *p* = 0.009), (c) a higher percentage of students from immigrant backgrounds in terms of children who were themselves born abroad (*t* = 9.28, *p* < 0.001), both of whose parents were not born in Germany (*t* = 3.59, *p* < 0.001) and who did not speak German at home (*t* = 3.59, *p* = 0.006), and (d) the percentage of students with special educational needs in general education schools (*t* = 2.01, *p* = 0.044). There were no statistically significant differences in grade retention, gender distribution, one parent being born abroad, or number of books at home across the two study cycles in 2016 and 2021 (see details in Appendix A).

### Does Student Reading Achievement in 2021 Differ From Pre-COVID-19 Times in 2016?

The average reading achievement in 2021 was 980 points. In 2016, fourth graders from the same schools had a mean reading achievement of 1,000 points. The gross model (Model 1) describes the difference in reading achievement between the study cycles without taking into account changes in student composition (see Table 1), but including school random intercepts. The fixed effect for the difference between the study cycles was 19 points (β*_cycle_* = −18.93, *SE* = 3.04, *p* < 0.001) for an average student in an average school. This difference of 19 points was statistically significant and corresponded to a standardized effect size of *d* = 0.19 (note that the SD is 100). The slight deviation from the average score difference (20 points) results from controlling for the random intercept. In conclusion, on average, students’ reading achievement was lower in 2021 than in 2016. This result supported our Hypothesis 1.

**Table 1:** Linear mixed-effect model explaining reading achievement.

	Model 1 gross study cycle difference (GM)		Model 2 net study cycle difference (NM)		Model 3 achievement gap differences (AM)	
	Estimate	*SE*	Estimate	*SE*	Estimate	*SE*
Intercept	**1001.32**	**4.39**	**1039.49**	**4.25**	**1044.04**	**5.13**
Study cycle (2016 = 0, 2021 = 1)	**−18.93**	**3.04**	**−13.80**	**3.03**	**−22.16**	**6.04**
Gender^a^ (male = 1, female = −1)			**−6.03**	**1.46**	**−6.54**	**2.05**
Relative cohort age (years)^*^			−2.95	5.73	−3.79	7.52
Enrolled (years)^*^			**−13.19**	**4.27**	**−13.78**	**5.49**
Retention (years)^*^			**−53.97**	**4.32**	**−50.20**	**6.67**
Child not born in Germany^+^			**−21.09**	**6.60**	−13.44	9.72
One parent not born in Germany^+^			**−17.78**	**4.62**	**−25.16**	**7.54**
Both parents not born in Germany^+^			**−31.51**	**4.78**	**−27.57**	**6.73**
German spoken at home^+^^,^^a^			**−10.20**	**5.86**	−13.18	7.06
Number of books at home^+^^,^^b^			**−36.16**	**3.73**	**−42.20**	**5.00**
Need for special education^+^^,^^c^			**−78.92**	**8.19**	**−88.00**	**16.47**
Year 21 x Gender					0.80	2.92
Year 21 x Age					1.19	11.28
Year 21 x Enrolled					0.68	8.45
Year 21 x Retention					−6.83	9.57
Year 21 x Child not born in Ger.					−10.17	13.76
Year 21 x One parent not born in Ger.					13.57	11.06
Year 21 x Both parents not born in Ger.					−7.70	9.93
Year 21 x German spoken at home					5.87	11.03
Year 21 x Number of books at home					11.25	6.68
Year 21 x Need for special education					14.11	21.43
Explained variance between schools	0.006		0.588		0.598	
Explained variance overall	0.010		0.167		0.168	

Study 2016 *N* = 2,208 and 2021 *N* = 2,082 with each *N* = 111 schools.

* Continuous variable centered.

SE, Standard error and Bold estimates: *p* < 0.05.

+ Dichotomous variables with dummy coding (0 vs. 1).

a Percentage of children who answered “I always speak German at home” or “almost always speak German at home.”

b Percentage of children who answered “Enough to fill two bookshelves (101–200)” or more.

c Children with an official diagnosis that justifies special educational needs (i.e., emotional disability).

### Does Student Reading Achievement in 2021 Differ From Pre-COVID-19 Times in 2016 When Adjusting for Student Composition?

The net model (Model 2) displays the difference in reading achievement between 2016 and 2021 adjusted for student composition. The net model displayed a significant effect of study cycle β*_cycle_* = −13.80, *SE* = 3.03, *p* < 0.001, indicating that the difference between 2016 and 2021 cannot fully be explained by the student composition variables. The corresponding effect size was *d* = 0.14. This supports H2 that average reading achievement declined from 2016 to 2021 even when adjusting for student composition. The mean expected reading achievement for 2016 given the student composition in 2016 is 1,000 (i.e., mean for 2016), while the mean expected reading achievement for 2021 given the student composition in 2021 is 980 (i.e., mean for 2021). However, we can estimate the expected mean reading achievement for 2021 based on the student composition for 2016. The expected mean reading achievement for 2021 given the student composition for 2016 is 986, and thus, 14 points (i.e., *d* = 0.14) lower than 2016.

In sum, these results indicate that the average reading achievement is lower in 2021 independently of student composition. This supports Hypothesis 2 that average reading achievement declined even when adjusting core characteristics of student composition.

### Are There Achievement Gaps Between Subgroups of Students and Did They Change Over Time?

Table 2 shows the estimated subgroup differences in reading achievement, achievement gaps, and changes in achievement gaps. Overall, the results suggest that the achievement gap between students born in Germany and students born in other countries widened from 2016 to 2021. The gap between students with both parents born in Germany and students with both parents born abroad tend to be larger in 2021 than it was in 2016. Similarly, the gaps between students who primarily spoke German at home and students who did not primarily speak German at home tended to widen. There was no increase in the gender gap between 2016 and 2021. Lastly, the gap between children with one parent born in another country and children with both parents born in Germany and children with more and less than 100 books seemed to close. However, none of these differences was statistically significant.

**Table 2:** Reading achievement gaps in different student subgroups.

Student subgroup	Reading achievement (*SE*)		Achievement Gap	Δ Gap
Gender	Girls	Boys		
2016	1,008 (4.4)	994 (4.9)	−14 (2.2)	2 (3.1)
2021	988 (5.3)	976 (6.5)	−12 (3.8)	
Country of birth (child)	Germany	Other		
2016	1,004 (4.2)	958 (10.1)	−46 (9.2)	−17 (11.4)
2021	991 (5.3)	928 (15.6)	−63 (14.7)	
Country of birth (one parent)	Germany	Other		
2016	1,004 (4.6)	987 (9.0)	−17 (7.8)	13 (11.4)
2021	983 (5.7)	979 (15.0)	−4 (13.8)	
Country of birth (both parents)	Germany	Other		
2016	1,010 (4.2)	971 (7.1)	−39 (5.8)	−16 (8.2)
2021	997 (5.5)	942 (11.4)	−55 (10.0)	
Language at home	German	Not German		
2016	1,008 (4.3)	975 (7.4)	−33 (6.0)	−8 (8.6)
2021	991 (5.6)	951 (12.0)	−41 (10.6)	
Books at home	More then 100	100 or less		
2016	1,034 (5.3)	985 (7.3)	−50 (5.0)	5 (6.8)
2021	1,012 (7.6)	967 (11.4)	−45 (10.6)	

Study 2016 *N* = 2,208 and 2021 *N* = 2,082 with each *N* = 111 schools.

The achievement gap model (Model 3) considers differential effects of the student composition variables. The model displays no significant interaction between the year and any of the student composition variables. This suggests that the achievement gaps in the student composition variables did not change significantly between 2016 and 2021. With respect to our hypotheses, we did find a gap between students with different socio-cultural capital, which is in accordance with H3.1.1. However, we did not find a widening gap between 2016 and 2021 (i.e., H3.1.2 was rejected). Furthermore, we found a gap between students from immigrant and non-immigrant backgrounds, which is in accordance with H3.2.1. However, we did not find a widening gap between 2016 and 2021 (i.e., H3.2.2 was rejected). Finally, we found a gender gap in reading achievement, which is in accordance with H3.3.1, but did not find a widening gap from 2016 to 2021 (i.e., H3.3.2 was rejected). In sum, none of the achievement gaps statistically significantly changed between 2016 and 2021.

## Discussion

The present work provided first empirical evidence on the status of reading achievement among German fourth graders after the COVID-19-related changes to schooling. Our study makes a cohort comparison of reading achievement among students from 111 elementary schools in Germany before the COVID-19 pandemic in 2016 and more than 1 year after the outbreak of the pandemic in 2021. We adjusted the results for student composition in both study cycles. In sum, there is clear evidence that reading achievement, a core learning outcome, is lower on average among current fourth graders compared to the pre-COVID-19 situation in 2016. The difference between 2016 and 2021 can only partially be explained by student composition. A difference of 19 points is way beyond changes in average reading achievement found in large-scale assessment over the past decades. Thus, it is likely that this decline in average reading achievement is at least partly due to COVID-19-related measures. The observed effects are in the range of the average impact of COVID-19-related school closures as reported in the meta-analysis by König and Frey (2022) (*d* = −0.18).

The observed decline in average reading achievement is remarkable. Baird and Pane (2019) discussed translating standardized effect sizes into years of learning to make them more interpretable. The average annual reading achievement gains in fourth grade are often considered *d* = 0.40 with a margin of error of ±0.06 (Hill et al., 2008). Thus, the decline of *d* = −0.19 means that fourth graders in 2021 are around half a year of learning behind fourth graders in 2016. The decrease of *d* = −0.14 when controlling for student composition would represent slightly more than 4 months of learning. Note that the effect size of annual literacy gains was not measured directly, and average annual literacy gains vary across studies (e.g., *d* = 0.29: Ditton and Krüsken, 2009; *d* = 0.48: Krüsken, 2007), so the half-year or 4-month learning time are not necessarily very precise estimates. Nonetheless, fourth graders in 2021 are substantially behind fourth graders in 2016, even with more conservative estimates. Hence, even though elementary schools implemented a variety of support measures during the COVID-19 pandemic (Huber et al., 2020; Lorenz et al., 2020; Meinck et al., 2022), the results presented here support the concern that younger students were particularly affected by the pandemic schooling situation (see also Tomasik et al., 2020).

Contrary to expectations, we did not find statistically significant effects indicating widening achievement gaps between subgroups of students—here: socio-cultural capital, immigration background, and gender. However, the statistical power for such interaction effects is limited in our study. Our study considered different sources of statistical uncertainty, plausible value variance, sampling variance, and imputation variance, as well as weighting, which imposed a high standard on finding significant changes in achievement gaps. There are recent findings from the German federal state Baden-Württemberg based on an annual population survey suggesting that schools with a large proportion of students with migration background and with lower average socio-cultural capital, respectively, had larger average losses in achievement than other schools (Schult et al., 2022). Therefore, it is likely that studies using larger samples or longitudinal designs can identify significant differences in achievement gaps. Thus, in light of the existing gaps and the low achievement levels of a substantial share of the student population, targeted support measures are clearly necessary. This finding is in line with previous studies (for Germany: Stanat et al., 2019, internationally: Mullis et al., 2017).

### Strengths and Limitations

There is a need for empirical evidence on the academic achievement of current student cohorts in order to understand how these students perform compared to their expected achievement in the absence of the COVID-19 pandemic. Our study is one of the first studies worldwide—and the first of its kind in Germany—to apply a rigorous methodology in order to estimate the actual status of students’ reading achievement in elementary schools. The presented analyses are based on a representative sample taking the standardized, well-established PIRLS reading achievement test. In contrast to other comparative studies, we present a school panel analysis. This has the main benefit of holding a number of key variables related to the educational environment, such as general school conditions (e.g., reading curricula) and school location, constant, allowing for a very high degree of comparability. Thus, the instrument and study design enable us to obtain reliable information on developments in achievement over time controlling for student composition as well as evidence on achievement gaps.

However, as a main limitation, it must be stated that no causal inferences on the effect of the containment measures during the COVID-19 pandemic on reading achievement since 2016 can be drawn. The prerequisites for causal inferences are not given. A control group is not available, since the COVID-19-related measures were applied to all schools, and our study is not longitudinal at the student level and therefore cannot control for pre-pandemic individual student characteristics. At least one of these two conditions (as well as a few others) would be necessary to estimate the causal effect of specific pandemic measures such as school closures of different lengths. In addition, there may be a slight underestimation of the full effect, as the measurement date in 2021 was on average 1 month later than in 2016.

Furthermore, we only investigated reading achievement as a comprehensive construct. However, reading is a multi-faceted construct (Graesser and McNamara, 2011) with many contributing subprocesses such as word recognition (e.g., decoding skills), language comprehension (e.g., verbal reasoning), and bridging processes (e.g., vocabulary knowledge) and additionally, active self-regulation, motivation, and engagement (Duke and Cartwright, 2021). All of these subprocesses could be influenced by the COVID-19 pandemic conditions. Further insights into which particular reading subprocesses were especially impaired could help to further improve post-COVID-19 reading interventions. We will have to leave this to further research, as the panel study was not originally designed to allow for these in-depth analyses.

### Implications: Research, Support, and Educational Policy

However, the presented findings lead to important conclusions regarding further research, educational practice, and educational policy. Further analyses may provide more in-depth insights. These include differentially considering reading achievement for literary texts compared with informational texts, which may lead to more gender-specific findings, as girls’ performance advantages at the end of fourth grade are especially prominent for literary texts (Mullis et al., 2017), and this may have been further reinforced by increased reading for pleasure during the COVID-19 pandemic-related restrictions. In addition, it should be examined whether the results also apply to other domains such as mathematics or to older groups of students. Finally, international comparisons are urgently needed to clarify whether the pattern found for Germany holds for other countries as well. This will be possible in the future using data from internationally comparative school achievement surveys such as PIRLS 2021 (elementary school, to be published in December 2022) and PISA 2022 (secondary schools, assessed in 2022). Similarly, national large-scale assessments of student achievement can also be insightful (Stanat et al., 2019) and could help to refine our findings in the future.

Regarding educational practice, it should be noted that compensatory measures have not been sufficiently effective for elementary school students in Germany more than a year after the onset of the COVID-19 pandemic-related restrictions on school operations but since then comprehensive measures have started to take place in Germany. Indeed, the findings highlight the need for comprehensive support—for all learners, as shown by the overall effect, but also targeted support for specific groups of students, as illustrated by the significant achievement gaps at the end of fourth grade, even if these were not further amplified compared to 2016. Here, coordinated targeted support approaches must be used that focus on systematically support reading skills in the classroom, extracurricular support during students’ leisure time, and during school vacations, as well as support from the family. Lastly, we assessed reading achievement shortly before most students in Germany transition to secondary schools. Therefore, the study provides information that could help secondary school teachers better understand the needs of rising fifth graders in post-COVID-19 times.

The findings are also informative for the design of educational policy. It should be concluded that the framework and conditions for learning in crisis situations need to be strengthened. This includes but is not limited to expanding the framework conditions and use of digital media, but also promoting resilience at all levels (i.e., among learners and their families, teachers, schools, and the educational system). Furthermore, self-regulated learning should be fostered among students of all ages, and last but not least, reading skills should be effectively supported at an early stage, as a key competency for all learners that enables them to acquire learning content relatively independently even in extraordinary learning situations such as distance learning.

The aim of the present study was to gain profound insights into the status of students’ achievement in the key competence of reading after a long period of COVID-19-related restrictions on learning at school and to identify any necessary support needs. In conclusion, society, as well as educational practice and educational policy more specifically, are now tasked with implementing effective supports for the children and adolescents affected by the COVID-19 pandemic in order to effectively secure their educational and life chances.

## Data Availability Statement

The datasets presented in this article are not readily available because publication restrictions apply until the end of 2022. When available, the datasets will be available here: https://www.fdz-bildung.de/home.

## Ethics Statement

Ethical review and approval was not required for the current study in accordance with the local legislation and institutional requirements. Written informed consent was not required in accordance with the national legislation and the institutional requirements. The original studies that led to the creation of the dataset were reviewed and approved by the Ministers of Education (“Kultusminister der Länder”) of all 16 federal states in Germany, and written informed consent to participate in these studies was provided by the participants’ legal guardian/next of kin.

## Author’s Note

Content, ethical aspects, and data protection have been thoroughly examined by the responsible (data protection) officers of each of the 16 German federal states.

## Author Contributions

NM and RL contributed to the conception and design of the study. UL prepared the database, performed the statistical analysis, and wrote the first draft of the method and result section. NM wrote the first draft of introduction and discussion. TS, RS, and RK wrote paragraphs of the manuscript. UL, NM, RL, TS, RS, RK, CK, and AF contributed to manuscript revision, read, and approved the submitted version. All authors contributed to the article and approved the submitted version.

## Funding

The study was funded by the Federal Ministry of Education and Research (BMBF) and the Standing Conference of the Ministers of Education and Cultural Affairs of the German federal states (KMK).

## Conflict of Interest

The authors declare that the research was conducted in the absence of any commercial or financial relationships that could be construed as a potential conflict of interest.

## Publisher’s Note

All claims expressed in this article are solely those of the authors and do not necessarily represent those of their affiliated organizations, or those of the publisher, the editors and the reviewers. Any product that may be evaluated in this article, or claim that may be made by its manufacturer, is not guaranteed or endorsed by the publisher.

## Appendix A

Descriptive results comparing the student composition in 2016 and 2021.

**Table:**

	Year	*M*	*SD*	Δ2021–2016	*t*	Mis (%)
Reading achievement	2016	1000.00 (5.29)	100			−
	2021	980.46 (5.32)	102	**−19.55 (3.08)**	−6.34	−
Relative cohort age (years)	2016	10.19 (0.01)	0.30			0.50
	2021	10.32 (0.01)	0.29	**0.13 (0.01)**	14.13	0.96
Late enrollment (years)	2016	0.03 (0.02)	0.43			0.50
	2021	0.06 (0.02)	0.43	**0.03 (0.01)**	2.59	0.96
Grade retention (years)	2016	0.13 (0.02)	0.35			0.50
	2021	0.13 (0.02)	0.37	0.01 (0.01)	0.77	0.98
Females (%)	2016	50.12 (0.95)				0
	2021	50.02 (1.05)		−0.10 (1.53)	−0.07	0
Not born in Germany						
Child (%)	2016	5.49 (0.65)				12.63
	2021	13.83 (1.53)		**8.34 (0.9)**	9.28	17.45
One parent (%)	2016	13.32 (1.10)				18.40
	2021	12.78 (1.09)		−0.53 (1.03)	−0.52	22.61
Both parents (%)	2016	22.01 (2.11)				18.41
	2021	26.72 (2.38)		**4.71 (1.31)**	3.59	22.61
German not spoken at home^a^ (%)	2016	18.86 (1.58)				10.58
	2021	22.26 (1.78)		**3.40 (1.23)**	2.76	13.04
Number of books at home (>100)^b^ (%)	2016	67.69 (1.69)				12.36
	2021	67.25 (1.62)		−0.44 (1.43)	−0.31	15.38
Special educational needs^c^ (%)	2016	2.98 (0.80)				1.96
	2021	4.12 (0.66)		**1.14 (0.57)**	2.01	0.00

Study 2016 *N* = 2,208 and 2021 *N* = 2,082, with *N* = 111 schools each. Bold estimates: *p* < 0.05

a Percentage of children selecting “I always speak German at home” or “almost always speak German at home.”

b Percentage of children selecting “Enough to fill two bookshelves (101–200)” or more.

c Children with an official diagnosis of special educational needs (i.e., emotional disability).

## References

[ref1] Ainley M. Hillman K. Hidi S. (2002). Gender and interest processes in response to literary texts: situational and individual interest. Learn. Instr. 12, 411–428. doi: 10.1016/S0959-4752(01)00008-1

[ref2] Baird M. D. Pane J. F. (2019). Translating standardized effects of education programs into more interpretable metrics. Educ. Res. 48, 217–228. doi: 10.3102/0013189X19848729

[ref3] Bates D. Mächler M. Bolker B. Walker S. (2014). Fitting linear mixed-effects models using lme4. arXiv [Preprint].

[ref4] Becker M. McElvany N. Kortenbruck M. (2010). Intrinsic and extrinsic reading motivation as predictors of reading literacy: a longitudinal study. J. Educ. Psychol. 102, 773–785. doi: 10.1037/a0020084

[ref5] Bell J. F. Sykes E. D. Vidal C. (2009). Birthdate Effects: A Review of the Literature From 1990-On. UK: University of Cambridge.

[ref7] Boudon R. (1974). Education, Opportunity, and Social Inequality: Changing Prospects in Western Society. Göttingen, Germany: Wiley.

[ref8] Bourdieu P. (1983). “Ökonomisches Kapital, kulturelles Kapital, soziales Kapital [Economic capital, cultural capital, social capital],” in Soziale Ungleichheiten [Social Unequalities]. ed. Kreckel R. (North-Holland: Schwartz), 183–198.

[ref10] Depping D. Lücken M. Musekamp F. Thonke F. (2021). “Kompetenzstände hamburger Schüler* innen vor und während der Corona-Pandemie [Competence levels of Hamburg students before and during the Corona pandemic],” in Schule während der Corona-Pandemie. Neue Ergebnisse Und Überblick über Ein Dynamisches Forschungsfeld [School during the Corona Pandemic. New Results and Overview of a Dynamic Field of Research]. eds. Fickermann D. Edelstein B. (Münster, Germany: Waxmann), 51–79.

[ref001] Destatis (2018). Allgemeinbildende Schulen - Fachserie 11 Reihe 1 – Schuljahr 2016 [General education schools - Section 11 Subsection 1 - Year 2016]. Statistisches Bundesamt. Available at: https://www.statistischebibliothek.de/mir/receive/DEHeft_mods_00071539

[ref002] Destatis (2021). Allgemeinbildende Schulen - Fachserie 11 Reihe 1 – Schuljahr 2021 [General education schools - Section 11 Subsection 1 - Year 2021]. Statistisches Bundesamt. Available at: https://www.statistischebibliothek.de/mir/receive/DEHeft_mods_00071539

[ref11] Ditton H. Krüsken J. (2009). Denn wer hat, dem wird gegeben werden? Eine Längsschnittstudie zur Entwicklung schulischer Leistungen und den Effekten der sozialen Herkunft in der Grundschulzeit. J. Educ. Res. Online 1, 33–61. doi: 10.25656/01:4555

[ref12] Dong C. Cao S. Li H. (2020a). Young children’s online learning during COVID-19 pandemic: Chinese parents’ beliefs and attitudes. Child Youth Serv. Rev. 118:105440. doi: 10.1016/j.childyouth.2020.105440, PMID: 32921857 PMC7476883

[ref13] Dong Y. Wu S. Dong W.-Y. Tang Y. (2020b). The effects of home literacy environment on children’s reading comprehension development: a meta-analysis. Educ. Sci.: Theory Pract. 20, 63–82. doi: 10.12738/jestp.2020.2.005

[ref14] Duke N. K. Cartwright K. B. (2021). The science of reading progresses: communicating advances beyond the simple view of reading. Read. Res. Q. 56, 25–44. doi: 10.1002/rrq.411

[ref15] Eagly A. H. Wood W. (1999). The origins of sex differences in human behavior: evolved dispositions versus social roles. Am. Psychol. 54, 408–423. doi: 10.1037/0003-066X.54.6.408

[ref003] Eickelmann B. Bos W. Gerick J. Goldhammer F. Schaumburg H. Schwippert K.. (eds.) (2019). ICILS 2018# Deutschland: Computer-und informationsbezogene Kompetenzen von Schülerinnen und Schülern im zweiten internationalen Vergleich und Kompetenzen im Bereich Computational Thinking [ICILS 2018# Germany: Computer and information-related competencies of students in the second international comparison and Computational Thinking competencies]. (Münster: Waxmann Verlag).

[ref16] Engzell P. Frey A. Verhagen M. D. (2021). Learning loss due to school closures during the COVID-19 pandemic. SocArXiv [Preprint]. doi: 10.31235/osf.io/ve4z7 PMC8092566 33827987

[ref17] Fitzgerald J. Elmore J. Koons H. Hiebert E. H. Bowen K. Sanford-Moore E. E.. (2015). Important text characteristics for early-grades text complexity. J. Educ. Psychol. 107, 4–29. doi: 10.1037/a0037289

[ref18] Gore J. Fray L. Miller A. Harris J. Taggart W. (2021). The impact of COVID-19 on student learning in New South Wales primary schools: an empirical study. Aust. Educ. Res. 48, 605–637. doi: 10.1007/s13384-021-00436-w, PMID: 33727761 PMC7952260

[ref19] Graesser A. C. McNamara D. S. (2011). Computational analyses of multilevel discourse comprehension. Top. Cogn. Sci. 3, 371–398. doi: 10.1111/j.1756-8765.2010.01081.x, PMID: 25164300

[ref20] Grätz M. Wiborg Ø. N. (2020). Reinforcing at the top or compensating at the bottom? Family background and academic performance in Germany, Norway, and the United States. Eur. Sociol. Rev. 36, 381–394. doi: 10.1093/esr/jcz069

[ref21] Grewenig E. Lergetporer P. Werner K. Woessmann L. Zierow L. (2020). COVID-19 and Educational Inequality: How School Closures Affect Low- and High-Achieving Students. CESifo Working Paper No. 8648. Munich: CESifo. Available at: https://www.cesifo.org/de/publikationen/2020/working-paper/COVID-19-and-educational-inequality-how-school-closures-affect-low 10.1016/j.euroecorev.2021.103920 PMC8474988 34602646

[ref22] Guo J. Marsh H. W. Parker P. D. Morin A. J. S. Yeung A. S. (2015). Expectancy-value in mathematics, gender, and socioeconomic background as predictors of achievement and aspirations: a multi-cohort study. Learn. Individ. Differ. 37, 161–168. doi: 10.1016/j.lindif.2015.01.008

[ref23] Hammerstein S. König C. Dreisörner T. Frey A. (2021). Effects of COVID-19-related school closures on student achievement: a systematic review. Front. Psychol. 12:746289. doi: 10.3389/fpsyg.2021.746289, PMID: 34603162 PMC8481663

[ref24] Hill C. J. Bloom H. S. Black A. R. Lipsey M. W. (2008). Empirical benchmarks for interpreting effect sizes in research. Child Dev. Perspect. 2, 172–177. doi: 10.1111/j.1750-8606.2008.00061.x

[ref25] Hochpöchler U. Schnotz W. Rasch T. Ullrich M. Horz H. McElvany N.. (2013). Dynamics of mental model construction from text and graphics. Eur. J. Psychol. Educ. 28, 1105–1126. doi: 10.1007/s10212-012-0156-z

[ref26] Huber S. G. Günther P. S. Schneider N. Helm C. Schwander M. Schneider J.. (2020). COVID-19 und aktuelle Herausforderungen in Schule und Bildung. Erste Befunde des Schul-barometers in Deutschland, Österreich und der Schweiz [COVID-19 and Current Challenges in Schools and Education. First Findings of the School Barometer in Germany, Austria and Switzerland] Münster, Germany: Waxmann.

[ref27] Huber S. G. Helm C. (2020). Lernen in Zeiten der Corona-Pandemie. Die Rolle familiärer Merkmale für das Lernen von Schüler*innen: Befunde vom Schul-Barometer in Deutschland, Österreich und der Schweiz [Learning in Times of the Corona Pandemic. The Role of Family Characteristics in Student Learning: Findings From the School Barometer in Germany, Austria, and Switzerland]. DDS – Die Deutsche Schule, Beiheft 16, 37–60.

[ref28] Hußmann A. Stubbe T. C. Kasper D. (2017). “Soziale Herkunft und Lesekompetenzen von Schülerinnen und Schülern [Social background and reading skills of students],” in IGLU 2016. Lesekompetenzen von Grundschulkindern in Deutschland im internationalen Vergleich [IGLU 2016: Reading Competencies of Primary School Children in Germany in International Comparison]. eds. Hußmann A. Wendt H. Bos W. Bremerich-Vos A. Kasper D. Lankes E.-M.. (Münster, Germany: Waxmann), 195–217.

[ref29] Hyde J. (2014). Gender similarities and differences. Annu. Rev. Psychol. 65, 373–398. doi: 10.1146/annurev-psych-010213-115057 23808917

[ref30] Kao G. Tienda M. (1995). Optimism and achievement: the educational performance of immigrant youth. Soc. Sci. Q. 76, 1–19.

[ref31] Kigel R. M. McElvany N. Becker M. (2015). Effects of immigrant background on text comprehension, vocabulary, and reading motivation: a longitudinal study. Learn. Instr. 35, 73–84. doi: 10.1016/j.learninstruc.2014.10.001

[ref32] Kim Y. S. G. (2020). Hierarchical and dynamic relations of language and cognitive skills to reading comprehension: testing the direct and indirect effects model of reading (DIER). J. Educ. Psychol. 112, 667–684. doi: 10.1037/edu0000407

[ref33] Kintsch W. (1988). The role of knowledge in discourse comprehension: a construction-integration model. Psychol. Rev. 95, 163–182. doi: 10.1037/0033-295X.95.2.163, PMID: 3375398

[ref34] Köhler C. Robitzsch A. Hartig J. (2020). A bias-corrected RMSD item fit statistic: an evaluation and comparison to alternatives. J. Educ. Behav. Stat. 45, 251–273. doi: 10.3102/1076998619890566

[ref35] König C. Frey A. (2022). The impact of COVID-19-related school closures on student achievement: a meta-analysis. Educ. Meas. Issues Pract. 41, 16–22. doi: 10.1111/emip.12495

[ref36] Kristen C. Dollmann J. (2012). “Migration und Schulerfolg: Zur Erklärung ungleicher Bildungsmuster [Immigration and school success: towards explaining unequal educational patterns],” in Handbuch Migration Und Bildung [Handbook Immigration and Education]. ed. Matzner M. (Weinheim, Germany: Beltz), 102–117.

[ref37] Krüsken J. (2007). “Entwicklung von Schülerleistungen und Zensuren in der Grundschule,” in Kompetenzaufbau und Laufbahnen im Schulsystem. ed. Ditton H. (Frankfurt, Germany: Waxmann), 41–62.

[ref38] Lepper C. Stang J. McElvany N. (2021). Gender differences in text-based interest: text characteristics as underlying variables. Reading Research Quarterly [Preprint]. doi: 10.1002/rrq.420

[ref39] Logan S. Johnston R. (2010). Investigating gender differences in reading. Educ. Rev. 62, 175–187. doi: 10.1080/00131911003637006

[ref40] Lohmar B. Eckhardt T. (2015). The Education System in the Federal Republic of Germany 2013/2014: A Description of the Responsibilities, Structures, and Developments in Education Policy for the Exchange of Information in Europe. Available at: https://www.kmk.org/fileadmin/Dateien/pdf/Eurydice/Bildungswesen-engl-pdfs/dossier_en_ebook.pdf

[ref41] Lorenz R. Brüggemann T. McElvany N. (2020). Unterricht während der Corona-Pandemie: Lehrkräftebefragung Ergebnisse Teil I “Der Unterricht” [Teaching During the Corona Pandemic: Teacher Survey Results Part I “The Lessons”]. Institut für Schulentwicklungsforschung der Technischen Universität Dortmund. Available at: https://www.tu-dortmund.de/storages/zentraler_bilderpool/user_upload/UCP_Kurzbericht_final.pdf

[ref42] Lorenz R. Yotyodying S. Eickelmann B. Endberg M. (2021). Schule digital – der Länderindikator 2021. Erste Ergebnisse und Analysen im Bundesländervergleich [Digital School: The State Indicator 2021. First Results and Analyses Comparing the Federal States]. Available at: https://eldorado.tu-dortmund.de/handle/2003/40566

[ref43] Lumley T. (2020). Survey: Analysis of Complex Survey Samples. R Package Version 4.0. 1–19. Available at: http://ftp.acc.umu.se/mirror/CRAN/web/packages/survey/survey.pdf

[ref44] Maldonado J. De Witte K. (2020). The effect of school closures on standardized student test outcomes. Br. Educ. Res. J. 48, 49–94. doi: 10.1002/berj.3754

[ref004] Martin M. Mullis I. Hooper M. (2017). Methoden und Verfahren in PIRLS 2016. International Association for the Evaluation of Educational Achievement. Chestnut Hill, USA: TIMSS & PIRLS International Study Center. Available at: https://files.eric.ed.gov/fulltext/ED580352.pdf

[ref45] McElvany N. Kessels U. Schwabe F. Kasper D. (2017). “Geschlecht und Lesekompetenz [Gender and reading competence],” in IGLU 2016. Lesekompetenzen von Grundschulkindern in Deutschland im internationalen Vergleich IGLU [2016: Reading Competencies of Primary School Children in Germany in International Comparison]. eds. Hußmann A. Wendt H. Bos W. Bremerich-Vos A. Kasper D. Lankes E.-M.. (Münster, Germany: Waxmann), 177–194.

[ref46] McElvany N. Lepper C. Lorenz R. Brüggemann T. (2021). “Unterricht während der Corona-Pandemie [Lessons during the corona pandemie],” in Generation Corona? Wie Jugendliche durch die Pandemie benachteiligt werden [How Are Young People Disadvantaged by Pandemic? How Young People Are Affected by Pandemic]. eds. Dohmen D. Hurrelmann K. (Weinheim: Beltz Juventa), 64–79.

[ref47] McElvany N. Schroeder S. Baumert J. Schnotz W. Horz H. Ullrich M. (2012). Cognitively demanding learning materials with texts and instructional pictures: teachers’ diagnostic skills, pedagogical beliefs, and motivation. Eur. J. Psychol. Educ. 27, 403–420. doi: 10.1007/s10212-011-0078-1

[ref48] Meinck S. Fraillon J. Strietholt R. (2022). The Impact of the COVID-19 Pandemic on Education. Interational Evidence From the Responses to Educational Disruption Survey (REDS). IEA. Available at: https://www.iea.nl/publications/international-evidence-responses-to-educational-disruption-survey

[ref49] Mo J. (2019). “How Does PISA Define and Measure Reading literacy?” PISA in Focus, No. 101, OECD Publishing, Paris.

[ref50] Monseur C. Adams R. (2009). Plausible values: how to deal with their limitations. J. Appl. Meas. 10, 1–15. 19671992

[ref51] Mullis I. Martin M. Foy P. Hooper M. (2017). PIRLS 2016. International Results in Reading. International Association for the Evaluation of Educational Achievement (IEA). Available at: https://files.eric.ed.gov/fulltext/ED580353.pdf

[ref52] Mullis I. Martin M. Sainsbury M. (2015). “PIRLS 2016 reading framework,” in PIRLS 2016 Assessment Framework. eds. Mullis I. Martin M. O. 2nd Edn. (Boston College, Chestnut Hill, MA: TIMSS & PIRLS International Study Center), 11–29.

[ref53] Ozuru Y. Dempsey K. McNamara D. S. (2009). Prior knowledge, reading skill, and text cohesion in the comprehension of science texts. Learn. Instr. 19, 228–242. doi: 10.1016/j.learninstruc.2008.04.003

[ref005] R Core Team (2020). R: A language and environment for statistical computing. Vienna, Austria: R Foundation for Statistical Computing Available at: https://www.R-project.org

[ref54] Reimers F. Schleicher A. (2020). Schooling Disrupted, Schooling Rethought. How the COVID-19 Pandemic is Changing Education. OECD. https://globaled.gse.harvard.edu/files/geii/files/education_continuity_v3.pdf

[ref55] Reiss K. Weis M. Klieme E. Köller O. (eds.) (2019). PISA 2018: Grundbildung Im Internationalen Vergleich [PISA 2018: Basic Education in International Comparison]. Münster, Germany: Waxmann Verlag.

[ref56] Robitzsch A. Grund S. Henke T. Robitzsch M. A. (2017). Package ‘Miceadds’. R Package: Madison, WI, USA. Available at: https://cran.r-project.org/web/packages/miceadds/miceadds.pdf

[ref57] Robitzsch A. Kiefer T. Wu M. (2019). TAM: Test Analysis Modules. R Package Version 3.3–10. https://CRAN.R-project.org/package=TAM

[ref58] Rose S. Twist L. Lord P. Rutt S. Badr K. Hope C.. (2021). Impact of School Closures and Subsequent Support Strategies on Attainment and Socio-Emotional Wellbeing in Key Stage 1: Interim Paper 1. Education Endowment Foundation (EEF). https://educationendowmentfoundation.org.uk/public/files/Publications/COVID-19_Resources/Impact_of_school_closures_KS1_interim_findings_paper_-_Jan_2021.pdf

[ref59] Rožman M. Meinck S. Chen M. (2022). “Impact of the pandemic on classroom teaching and learning,” in The Impact of the COVID-19 Pandemic on Education. International evidence from the Response to Education Disruption Survey (REDS). eds. Meinck S. Fraillon J. Strietholt R. (Paris, France: United Nations Educational, Scientific and Cultural Organization (UNESCO)), 54–83.

[ref60] Sánchez Amate J. J. Luque de la Rosa A. Gutiérrez Cáceres R. Vargas Serrano A. (2021). The effects of COVID-19 in the learning process of primary school students: a systematic review. Educ. Sci. 11:654. doi: 10.3390/educsci11100654

[ref61] Savolainen H. Ahonen T. Aro M. Tolvanen A. Holopainen L. (2008). Reading comprehension, word reading and spelling as predictors of school achievement and choice of secondary education. Learn. Instr. 18, 201–210. doi: 10.1016/j.learninstruc.2007.09.017

[ref62] Schneider S. Beege M. Nebel S. Schnaubert L. Rey G. D. (2021). The cognitive-affective-social theory of learning in digital environments (CASTLE). Educ. Psychol. Rev. 34, 1–38. doi: 10.1007/s10648-021-09626-5, PMID: 34226808 PMC8242289

[ref006] Schult J. Mahler N. Fauth B. Lindner M. A. (2021). Did students learn less during the COVID-19 pandemic? Reading and mathematics competencies before and after the first pandemic wave. [Preprint]. doi: 10.31234/osf.io/pqtgf

[ref63] Schult J. Mahler N. Fauth B. Lindner M. A. (2022). Long-term consequences of repeated school closures during the COVID-19 pandemic for reading and mathematics competencies. Psyarxiv [Preprint] doi: 10.31234/osf.io/dfqeg

[ref64] Schunk D. H. Zimmerman B. J. (2006). Influencing Children’s self-efficacy and self-regulation of reading and writing through modeling. Read. Writ. Q. 23, 7–25. doi: 10.1080/10573560600837578

[ref65] Schwippert K. (2019). Was wird aus den Büchern? Sozialer Hintergrund von Lernenden und Bildungsungleichheit aus Sicht der international vergleichenden Erziehungswissenschaft. J. Educ. Res. Online 11, 92–117. doi: 10.25656/01:16789

[ref66] Sénéchal M. LeFevre J. A. (2002). Parental involvement in the development of children’s reading skill: a five-year longitudinal study. Child Dev. 73, 445–460. doi: 10.1111/1467-8624.00417, PMID: 11949902

[ref67] Stanat P. Schipolowski S. Mahler N. Weirich S. Henschel S. (2019). IQB-Bildungstrend 2018: Mathematische Und Naturwissenschaftliche Kompetenzen Am Ende der Sekundarstufe I Im Zweiten Ländervergleich [IQB Education Trend 2018: Mathematics and Science Competencies at the End of Secondary School Level I in the Second Country Comparison]. Münster, Germany: Waxmann.

[ref68] Steinmayr R. Lazarides R. Weidinger A. Christiansen H. (2021). Teaching and learning during the first COVID-19 school lockdown: realization and associations with parent-perceived students’ academic outcomes. Zeitschrift für Padagogische Psychol. 35, 85–106. doi: 10.1024/1010-0652/a000306

[ref69] Storey N. Zhang Q. (2021). A meta-analysis of COVID learning loss. EdArXiv [Preprint] doi: 10.35542/osf.io/qekw2

[ref70] Tomasik M. J. Helbling L. A. Moser U. (2020). Educational gains of in-person vs. distance learning in primaryelementary and secondary schools: a natural experiment during the COVID-19 pandemic school closures in Switzerland. Int. J. Psychol. 56, 566–576. doi: 10.1002/ijop.12728, PMID: 33236341 PMC7753520

[ref71] Toste J. R. Didion L. Peng P. Filderman M. J. McClelland A. M. (2020). A meta-analytic review of the relations between motivation and reading achievement for K–12 students. Rev. Educ. Res. 90, 420–456. doi: 10.3102/0034654320919352

[ref72] Van der Linden W. J. (2016). “Unidimensional logistic item response models,” in Handbook of Item Response Theory. (New York, USA: CRC Press), 13–30.

[ref73] Voogt J. Roblin N. P. (2012). A comparative analysis of international frameworks for 21st century competences: implications for national curriculum policies. J. Curric. Stud. 44, 299–321. doi: 10.1080/00220272.2012.668938

[ref74] Wang L. Finch H. (2018). Motivation variables mediate the relationship between socioeconomic status and academic achievement. Psychol. Educ. 55, 123–136. doi: 10.1002/per.842

[ref75] Wang Z. Sabatini J. O’Reilly T. Weeks J. (2019). Decoding and reading comprehension: a test of the decoding threshold hypothesis. J. Educ. Psychol. 111, 387–401. doi: 10.1037/edu0000302

[ref76] Wendt H. Schwippert K. (2017). “Lesekompetenzen von Schülerinnen und Schülern mit und ohne Migrationshintergrund [Reading competencies of students with and without a immigration background],” in IGLU 2016. Lesekompetenzen von Grundschulkindern in Deutschland im internationalen Vergleich. eds. Hußmann A. Wendt H. Bos W. Bremerich-Vos A. Kasper D. Lankes E.-M.. (Münster, Germany: Waxmann), 219–234.

[ref77] Werner K. Woessmann L. (2021). The legacy of covid-19 in education. CESifo Working Paper, No. 9358, Center for Economic Studies and ifo Institute (CESifo). Available at: http://hdl.handle.net/10419/248903

[ref78] Wigfield A. Eccles J. (2000). Expectancy–value theory of achievement motivation. Contemp. Educ. Psychol. 25, 68–81. doi: 10.1006/ceps.1999.1015 10620382

[ref79] Wigfield A. Gladstone J. Turci L. (2016). Beyond cognition: reading motivation and reading comprehension. Child Dev. Perspect. 10, 190–195. doi: 10.1111/cdep.12184, PMID: 27617030 PMC5014370

[ref80] Woessmann L. Freundl V. Grewenig E. Lergetporer P. Werner K. Zierow L. (2020). Bildung in der Coronakrise: Wie haben die Schulkinder die Zeit der Schulschließungen verbracht, und welche Bildungsmaßnahmen befürworten die Deutschen? [“Education in the Corona Crisis: How Have Schoolchildren Spent the Period of School Closures, and What Educational Measures do German’s Advocate?”], ifo Schnelldienst, 73. Available at: https://www.ifo.de/DocDL/sd-2020-09-woessmann-etal-bildungsbarometer-corona.pdf

[ref81] Wrase M. (2020). “Schulrechtliche Herausforderungen in Zeiten der Corona-Pandemie [School law challenges in times of the Corona pandemic],” in “Langsam vermisse ich die Schule…” – Schule während und nach der Corona-Pandemie. eds. Fickermann D. Edelstein B. (Münster, Germany: Waxmann), 105–116.

[ref82] Zierer K. (2021). Effects of pandemic-related school closures on pupils’ performance and learning in selected countries: a rapid review. Educ. Sci. 11:252. doi: 10.3390/educsci11060252

